# Nephrotoxicity of cisplatin combination chemotherapy in thoracic malignancy patients with CKD risk factors

**DOI:** 10.1186/s12885-016-2271-8

**Published:** 2016-03-15

**Authors:** Ko Sato, Satoshi Watanabe, Aya Ohtsubo, Satoshi Shoji, Daisuke Ishikawa, Tomohiro Tanaka, Koichiro Nozaki, Rie Kondo, Masaaki Okajima, Satoru Miura, Junta Tanaka, Takuro Sakagami, Toshiyuki Koya, Hiroshi Kagamu, Hirohisa Yoshizawa, Ichiei Narita

**Affiliations:** Department of Medicine (II), Niigata University Medical and Dental Hospital, Niigata City, Niigata Japan; Bioscience Medical Research Center, Niigata University Medical and Dental Hospital, Niigata City, Niigata Japan; Department of Health Promotion Medicine, Niigata University Medical and Dental Hospital, Niigata City, Niigata Japan

**Keywords:** Cisplatin, Nephrotoxicity, Chronic kidney disease, Acute kidney injury

## Abstract

**Background:**

Nephrotoxicity is the major side effect that limits the dose of cisplatin that can be safely administered, and it is a clinical problem in cancer patients who received cisplatin combination chemotherapy. Recent evidence has demonstrated that patients with chronic kidney disease (CKD) have an increased risk of developing acute kidney injury (AKI). The present study was conducted to evaluate the prevalence of CKD risk factors in patients who received cisplatin and to assess the correlation between CKD risk factors and cisplatin-induced AKI.

**Methods:**

We retrospectively analyzed 84 patients treated with cisplatin combination chemotherapy for thoracic malignancies. AKI was defined as a decrease in the estimated glomerular filtration rate (eGFR) > 25 % from base line, an increase in the serum creatinine (sCre) level of > 0.3 mg/dl or ≥ 1.5 times the baseline level.

**Results:**

Eighty of the 84 patients (95.2 %) had at least one risk factor for CKD. All enrolled patients received cisplatin with hydration, magnesium supplementation and mannitol. Cisplatin-induced AKI was observed in 18 patients (21.4 %). Univariate analysis revealed that cardiac disease and use of non-steroidal anti-inflammatory drugs (NSAIDs) were associated with cisplatin-induced nephrotoxicity (odds ratios [OR] 6 and 3.56, 95 % confidence intervals [CI] 1.21–29.87 and 1.11–11.39, *p* = 0.04 and *p* = 0.04, respectively). Multivariate analysis revealed that cisplatin nephrotoxicity occurred significantly more often in patients with both risk factors (OR 13.64, 95 % CI 1.11–326.83, *p* = 0.04). Patients with more risk factors for CKD tended to have a greater risk of developing cisplatin-induced AKI.

**Conclusions:**

We should consider avoiding administration of cisplatin to patients with CKD risk factors, particularly cardiac disease and NSAID use.

**Electronic supplementary material:**

The online version of this article (doi:10.1186/s12885-016-2271-8) contains supplementary material, which is available to authorized users.

## Background

Cisplatin is one of the most commonly administered agents in the treatment of cancer. Cisplatin-based chemotherapy has demonstrated survival benefits for patients with resected early-stage non-small cell lung cancer (NSCLC), advanced-stage NSCLC, small-cell lung cancer (SCLC) and malignant pleural mesothelioma [1–4]. However, cisplatin can have severe side effects—in particular, cisplatin-induced nephrotoxicity, which is dose dependent and is the major side effect that limits the acceptable dose of cisplatin [5]. Impairment of renal function has been shown to be a clinical problem in 25–35 % of patients treated with cisplatin [5]. Therefore, it is important to identify the predictors of cisplatin-induced nephrotoxicity and to avoid administrating cisplatin to patients with these risk factors.

Recent studies have reported several risk factors for chronic kidney disease (CKD), including lifestyle-related diseases, smoking, use of non-steroidal anti-inflammatory drugs (NSAIDs), cardiac disease and cerebrovascular disease [6]. Patients with risk factors for CKD may have a higher incidence of cisplatin-induced nephrotoxicity. Indeed, acute kidney injury (AKI) is more frequently observed in patients with CKD [7, 8]. Furthermore, recent studies have reported that patients with lung cancer have a higher incidence of comorbidities than do patients with other cancers [9]. The aims of this study were to evaluate the prevalence of CKD risk factors in thoracic malignancy patients who received cisplatin and to elucidate the correlation between nephrotoxicity due to cisplatin-based chemotherapy in patients with CKD risk factors.

## Methods

### Patients

We retrospectively analyzed the data of consecutive patients who received cisplatin combination chemotherapy for thoracic malignancies as the first-line chemotherapy in our hospital between January 2006 and December 2012. All patients provided written informed consent. The study was conducted in accordance with the Helsinki Declaration of the World Medical Association. The protocol was approved by the institutional review board of Niigata University.

### Hydration and treatment methods

Patients received cisplatin combination chemotherapy every 3–4 weeks for 4–6 cycles. As a common antiemetic premedication, granisetron (3 mg) or palonosetron (0.75 mg) was combined with dexamethasone (9.9 mg) and dissolved in 100 ml of normal saline and infused to the patient, and oral aprepitant (125 mg on day1, 80 mg on days 2–3) was given before and after chemotherapy. Cisplatin was administered in 250 mL of normal saline solution over 2 h in combination with other chemotherapeutic agents and 3000–3850 ml of hydration. All patients received 40 mEq of magnesium sulfate and 300 ml of mannitol to prevent nephrotoxicity (Additional file 1: Figure S1).

### Nephrotoxicity evaluation

The estimated glomerular filtration rate (eGFR) (mL/min/1.73 m^2^) was calculated using the equation of the Japanese Society of Nephrology: eGFR (mL/min/1.73 m^2^) = 194 x serum creatinine (sCre)^-1.094^ × age^-0.287^ (×0.739 if female) [10]. We calculated and recorded the eGFR at four time points: before the administration of cisplatin combination chemotherapy, the minimum value during chemotherapy, 4 weeks after the last administration of cisplatin and the maximum value during the follow-up period (6 months after the last administration of cisplatin). AKI was defined as follows: a decrease in eGFR > 25 % of baseline, an increase in sCre level of > 0.3 mg/dl or ≥ 1.5 times the baseline level in reference to the RIFLE (risk, injury, failure, loss, and end stage) criteria and the Common Terminology Criteria for Adverse Events version 4.0 [11, 12].

### Risk factors

To identify the risk factors potentially associated with the occurrence of a cisplatin-induced nephrotoxicity, we examined the relationship between AKI incidence and the following 17 CKD risk factors: old age (≥70 years old), proteinuria, hematuria, dyslipidemia, diabetes mellitus, hypertension, hyperuricemia, obesity (body mass index ≥ 25), smoking, NSAID use, cerebrovascular disease, cardiac disease, autoimmune disease, infectious disease, urinary-tract stone and a history of acute renal failure [13]. We also assessed the following potential risk factors: hypoalbuminemia (serum albumin level < 3.5 g/dL), Eastern Cooperative Oncology Group performance status (PS ≥ 1), the number of CKD risk factors, sex, Charlson Comorbidity Index (CCI) score, and combined anticancer drugs [14–16].

### Statistical analysis

The differences in the categorical variables between patients with and those without AKI were determined using the chi-squire test or Fisher’s exact test, as appropriate. Two-sided *P* < 0.05 was considered statistically significant. To identify the variables significantly associated with AKI, multivariate logistic regression analysis was performed. All statistical analyses were performed using the JMP 9.0.2 statistical software (SAS Institute, Cary, NC, USA).

## Results

### Patient characteristics

A total of 84 patients who were treated with cisplatin combination chemotherapy for thoracic malignancies were included in this study. The patient characteristics are listed in Table 1. There were 19 (22.6 %) female patients and the median age of all the patients was 61 years old (range, 33–75 years). Seventy-nine (94 %) patients had a PS 0–1, 52 (61.9 %) had NSCLC and 19 (22.6 %) had SCLC. The median cisplatin dosage was 80 mg/m^2^/day, and the median number of cycles was 4 (range, 1–6). The patients received various cisplatin combination drugs, including vinorelbine in 26 (31 %), etoposide in 14 (16.7 %), docetaxel in 12 (14.3 %), pemetrexed in 11 (13.1 %), gemcitabine in 4 (4.8 %), S-1 in 4 (4.8 %), irinotecan in 2 (2.4 %), and others in 11 (13.1 %). The median CCI was 6 (range, 2–10).

**Table 1:** Base line characteristics of all study patients (*n* = 84)

Parameter	
Gender	
Male	65 (77.3 %)
Female	19 (22.6 %)
Age (y)	
Median (Range)	61 (33–75)
Performance status	
0/1/2	41/38/0
3/4/unknown	1/0/4
Histology	
Small cell carcinoma	19 (22.6 %)
Adenocarcinoma	37 (44 %)
Large-cell carcinoma	2 (2.4 %)
Squamous cell carcinoma	13 (15.5 %)
Malignant pleural mesothelioma	6 (7.1 %)
Thymic malignancy	2 (2.4 %)
Others	5 (6 %)
Cisplatin	
Median dose (mg/m^2^/day) (range)	80 (40–80)
Median cycle (range)	4 (1–6)
Anticancer drugs	
Vinorelbine	26 (31 %)
Etoposide	14 (16.7 %)
Docetaxel	12 (14.3 %)
Pemetrexed	11 (13.1 %)
Gemcitabine	4 (4.8 %)
S-1	4 (4.8 %)
Irinotecan	2 (2.4 %)
Others	11 (13.1 %)
Serum creatinine (mg/dl)	
Median (range)	0.72 (0.50–0.99)
eGFR (mL/min/1.73 m^2^)	
Median (range)	81.1 (59.2–113.0)
Charlson Comorbidity Index	
Median (range)	6 (2–10)

### Incidence and outcome of AKI

Cisplatin-induced AKI was observed in 18 (21.4 %) of the 84 enrolled patients (Additional file 2: Table S1). In the AKI group and in the no AKI group, the median eGFR before administration of cisplatin was 79.2 (range, 59.2–101) and 82.4 (range, 60–113) mL/min/1.73 m^2^, respectively. Two patients had mild renal dysfunction before cisplatin administration (eGFR, 59.2 and 59.6 mL/min/1.73 m^2^). Additional file 2: Table S1 shows the number of patients with risk factors for CKD in the AKI group and the no AKI group. In both groups, the most frequent risk factor was smoking (77.8 % in the patients with AKI and 83.3 % in the patients without AKI). All 18 (100 %) of the patients with AKI and 61 (92.4 %) of the patients without AKI had at least one risk factor.

**Fig. 1:**
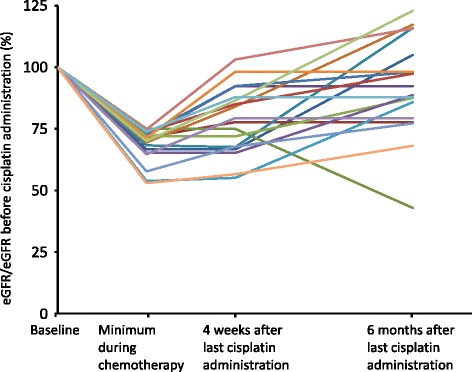
Changes of estimated glomerular filtration rate (eGFR). The eGFR was measured at four time points; before the treatment, under treatment, 4 weeks after last cisplatin administration and during follow-up period (6 months after last cisplatin administration)

Figure 1 shows the changes in eGFR in the 18 patients with AKI. During follow-up period (6 months after the last administration of cisplatin), the eGFR in 5 patients (27.8 %) recovered to baseline, the eGFR in 11 patients (61.1 %) recovered to between 75 % and baseline, and the eGFR in 2 patients (11.1 %) did not recover to 75 % of baseline.

### Risk factors for developing AKI

In the univariate analysis, we identified cardiac disease (odds ratio [OR] 6, 95 % confidence interval [CI] 1.21–29.87, *p* = 0.04) and NSAID use (OR 3.56, 95 % CI 1.11–11.39, *p* = 0.04) as significant predictors of AKI (Table 2). The eGFR before treatment (*p* = 0.22), and the other risk factors for CKD, such as PS ≥ 1 (*p* = 0.48), hypoalbuminemia (*p* = 0.49), female sex (*p* = 0.56), CCI score (*p* = 0.78), the type of anticancer drugs (*p* = 0.15–0.9) and the number of treatment cycles (*p* = 0.74) were not significantly associated with cisplatin-induced AKI. Next, we included cardiac disease and NSAID use in the logistic regression model with the addition of age and sex to assess the contribution of each risk factor to cisplatin-induced AKI. Although each of these factors was not a significant predictor of cisplatin-induced AKI, we found that cardiac disease and NSAID use had relatively higher odds ratios (OR 5, 95 % CI 0.89–30.4 and OR 3.45, 95 % CI 0.97–12.12, respectively; Table 3). Additionally, AKI occurred significantly more often in patients who presented both cardiac disease and NSAID use (OR 13.64, 95 % CI 1.11–326.83, *p* = 0.04). The multivariate analyses also revealed that the patients with more risk factors for CKD tended to have an increased risk of developing cisplatin-induced AKI.

**Table 2:** Univariate analysis by chi-square test or Fisher’s exact test

Risk factor	Odds ratio	95 % C.I.	*P*-value
CKD risk factors			
Smoking	0.7	0.19–2.53	0.59
Hypertension	2.3	0.79–6.66	0.12
Dyslipidemia	2.25	0.70–7.20	0.17
Use of NSAIDs	3.56	1.11–11.39	**0.04**
Obesity	0.51	0.10–2.50	0.4
Diabetes mellitus	0.91	0.17–4.70	0.91
Hyperuricemia	1.05	0.20–5.57	0.95
Urinary tract stone	-		0.12
Age ≥70	0.5	0.06–4.31	0.52
Cardiac disease	6	1.21–29.87	**0.04**
Hematuria	-		0.29
Cerebrovascular disease	-		0.29
Autoimmune disease	4	0.52–30.61	0.15
Proteinuria	-		0.6
Infectious disease	-		-
Past history of ARF	-		-
Pt. with CKD risk	-		0.29
Pt. without CKD risk	-		0.29
No. of CKD risk factors			
≤2	1		
3	1.54	0.39–5.96	0.72
4	1.5	0.34–6.7	0.69
≥5	5	0.85–29.39	0.09
Other risk factors			
PS ≥1	0.68	0.23–2.01	0.48
Alb <3.5 g/dL	0.69	0.24–1.97	0.49
Female	1.43	0.44–4.69	0.56
Anticancer drugs			
Vinorelbine	1	0.35–3.25	0.9
Etoposide	2.44	0.70–8.49	0.15
Docetaxel	0.7	0.14–3.53	0.66
Pemetrexed	0.33	0.04–2.76	0.29
Gemcitabine	3.94	0.51–30.14	0.16
S-1	3.94	0.51–30.14	0.16

*AKI* acute kidney injury, *CKD* chronic kidney disease, *NSAID* non-steroidal anti-inflammatory drug, *ARF* acute renal failure, *PS* performance status, *C.I.* confidential interval

Bold *P*-values are significant at the 0.05 level

**Table 3:** Multivariate logistic regression analysis adjusted for age and sex

Parameter	Odds ratio	95 % C.I.	*P*-value
Age	1.01	0.95–1.08	0.74
Female	1.99	0.52–7.11	0.3
Cardiac disease	5	0.89–30.4	0.07
Use of NSAIDs	3.45	0.97–12.12	0.06
Cardiac disease and use of NSAIDs	13.64	1.11–326.83	**0.04**
No. of CKD risk factors			
≥3	2.11	0.69–6.79	0.19
≥4	2.68	0.83–8.5	0.1
≥5	4.55	0.73–28.65	0.1

*NSAID* non-steroidal anti-inflammatory drug

Bold *P*-value is significant at the 0.05 level

## Discussion

Cisplatin is one of the most active cytotoxic agents in treatment of thoracic malignancies. Cisplatin or carboplatin combination chemotherapy is recommended as the first-line chemotherapy for patients with lung cancer [17, 18]. Because cisplatin has more severe non-hematological toxicity than carboplatin does, particularly in terms of nephrotoxicity and gastrointestinal toxicity, cisplatin is more poorly tolerated than carboplatin. Although cisplatin-based chemotherapy has been reported to be superior to carboplatin-based chemotherapy in non-squamous NSCLC patients, the number of patients administered cisplatin-based chemotherapy was limited in clinical practice [2]. Recent progress in antiemetic therapies has greatly reduced cisplatin-induced gastrointestinal toxicity. We and other investigators have reported that triple antiemetic therapy with a 5-HT_3_ receptor antagonist, dexamethasone and aprepitant ameliorates cisplatin-induced nausea and vomiting [19, 20]. However, cisplatin-induced nephrotoxicity remains an unsolved problem. Although previous studies have reported less toxic methods for administrating cisplatin, such as hydration with the supplementation of magnesium and mannitol, cisplatin-induced nephrotoxicity still occurs in 6–14 % of patients [21, 22]. Compatible with these studies, the current study showed that the incidence of AKI was 21.4 % (18/84), despite the administration of hydration, magnesium supplementation and mannitol. We need to develop more effective strategies to prevent cisplatin-induced nephrotoxicity.

Previous studies demonstrated that old age, female sex, current smoking, hyperuricemia, hypoalbuminemia, diabetes mellitus, cardiovascular disease and NSAID use are risk factors for cisplatin-induced nephrotoxicity [15, 21, 23]. Because recent evidence has demonstrated that CKD is a risk factor for AKI, we hypothesized that patients with risk factors for CKD would also be at risk for cisplatin-induced nephrotoxicity [7]. The present study showed that comorbidities, including CKD risk factors, were frequent in patients with thoracic malignancies (Additional file 2: Table S1). Univariate analysis demonstrated that cardiac disease and NSAID use were statistically significant predictors of cisplatin-induced nephrotoxicity (Table 2). Although multivariate analysis revealed that no single factor was significantly associated with cisplatin-induced AKI, AKI did occur significantly more frequently in patients who had both cardiac disease and NSAID use (Table 3). The risk factors for cardiac disease, such as hypertension, diabetes mellitus, dyslipidemia and smoking, are also risk factors for CKD. These factors cause atherosclerosis in the renal arteries and induce chronic ischemic injury to the tubulointerstitium [24]. Because tubular cells are the major target of cisplatin-induced nephrotoxicity, it is not surprising that cardiac disease is associated with this complication [25]. Nonselective cyclooxygenase (COX) inhibition by NSAIDs decreases prostaglandin synthesis and induces vasoconstriction and renal ischemia. Cisplatin has been suggested to induce injury in the renal vasculature and ischemic injury of the kidneys; thus these effects of NSAIDs may exacerbate cisplatin-induced nephrotoxicity [25]. In this study, the non-selective COX inhibitor loxoprofen was prescribed to 13 of the 17 patients who had received NSAIDs (76.8 %). Use of selective COX 2 inhibitors may reduce the risk of cisplatin-induced nephrotoxicity. Furthermore, our study showed that an increased number of CKD risk factors may be related to a higher incidence of cisplatin-induced AKI (Tables 2 and 3). Patients without CKD risk factors did not develop cisplatin-induced AKI. By contrast, patients with 5 or more CKD risk factors had relatively higher odds ratio (OR 4.55, 95 % CI 0.73–28.65; Table 3). We also investigated whether CCI score, which is a method of classifying prognostic comorbidity, correlated with the incidence of cisplatin-induced AKI, but no association was found [16].

In previous studies, cisplatin-induced nephrotoxicity has been assessed on the basis of the elevated sCre levels [21, 26]. However, Launay-Vacher et al. analyzed data for 4684 cancer patients and found that elevated sCre levels were less sensitive than was decreased Cre clearance for detecting renal impairment [27]. In that study, renal impairment was commonly found in cancer patients (57.4 %), and 53.4 % required dose reduction due to nephrotoxicity. The authors concluded that the use of nephrotoxic drugs should be avoided in cancer patients at high risk for AKI. Thus, we included decreased eGFR (estimated using the equation developed by the Japanese Society of Nephrology) in our AKI criteria [10]. The current study demonstrated that a decrease in the eGFR ≥ 25 % from the baseline level was detected in 22.4 % (18/84) of the patients, although only 9.5 % (8/84) exhibited elevated sCre levels of > 0.3 mg/dl or ≥ 1.5 times baseline. These data suggest that GFR should be included in assessment of cisplatin-induced nephrotoxicity. Cisplatin causes cell injury and death in the proximal and distal tubules. Although the long-term effects of cisplatin on renal function have not been fully elucidated, previous studies indicated that cisplatin treatment may lead to subclinical but permanent reductions in GFR [28]. By contrast, Moon et al. reported that the elevated sCre levels recovered in 80.5 % of patients after cisplatin-induced AKI [29]. In the present study, of the 18 patients who had cisplatin-induced AKI, the eGFR in 16 (88.9 %) recovered to more than 75 % of the baseline level during the follow-up period. Cisplatin combination chemotherapy has been recommended as the first-line chemotherapy for lung cancer patients [17, 18]. Patients who develop cisplatin-induced AKI may have a higher risk of nephrotoxicity with subsequent chemotherapies. Indeed, AKI is a well-defined risk factor for CKD, and patients who develop AKI have a several-fold increased risk of developing CKD [30]. Further investigation should be carried out to evaluate whether subsequent chemotherapies can be safely administered to patients who had experienced cisplatin AKI.

## Conclusion

To our knowledge, this is the first study to assess the incidence of cisplatin-induced nephrotoxicity in patients with CKD risk factors. We identified cardiac disease and NSAID use as risk factors for cisplatin-induced nephrotoxicity. In addition, patients who had a higher number of CKD risk factors tended to have an increased risk of cisplatin-induced nephrotoxicity.

## Availability of data and materials

The dataset supporting the conclusions of this article is included within the Additional file 3: Dataset S1.

## Additional files

Additional file 1: Figure S1. Chemotherapy hydration protocol used in this study. Cisplatin and other anticancer drugs were administrated with 3850 ml of hydration, magnesium sulfate (40 mEq) and mannitol (300 ml). (PPTX 79 kb) Additional file 2: Table S1. The characteristics of the patients with or without AKI. (XLSX 14 kb) Additional file 3: Dataset S1. The characteristics of the patients. (XLSX 28 kb)

## Abbreviations

AKI: acute kidney injury
CCI: Charlson Comorbidity Index
CKD: chronic kidney disease
COX: cyclooxygenase
eGFR: estimated glomerular filtration rate
NSAIDs: non-steroidal anti-inflammatory drugs
NSCLC: non-small cell lung cancer
SCLC: small-cell lung cancer
sCre: serum creatinine
