# Supplementary material for: Nephrotoxicity of cisplatin combination chemotherapy in thoracic malignancy patients with CKD risk factors
Source: BMC Cancer. 2016 Mar 15;16:222. doi: 10.1186/s12885-016-2271-8 (PMC4793503; doi:10.1186/s12885-016-2271-8)
Supplement: Additional file 1: Figure S1. — Chemotherapy hydration protocol used in this study. Cisplatin and other anticancer drugs were administrated with 3850 ml of hydration, magnesium sulfate (40 mEq) and mannitol (300 ml). (PPTX 79 kb) [file 12885_2016_2271_MOESM1_ESM.pptx]

## Slide 1
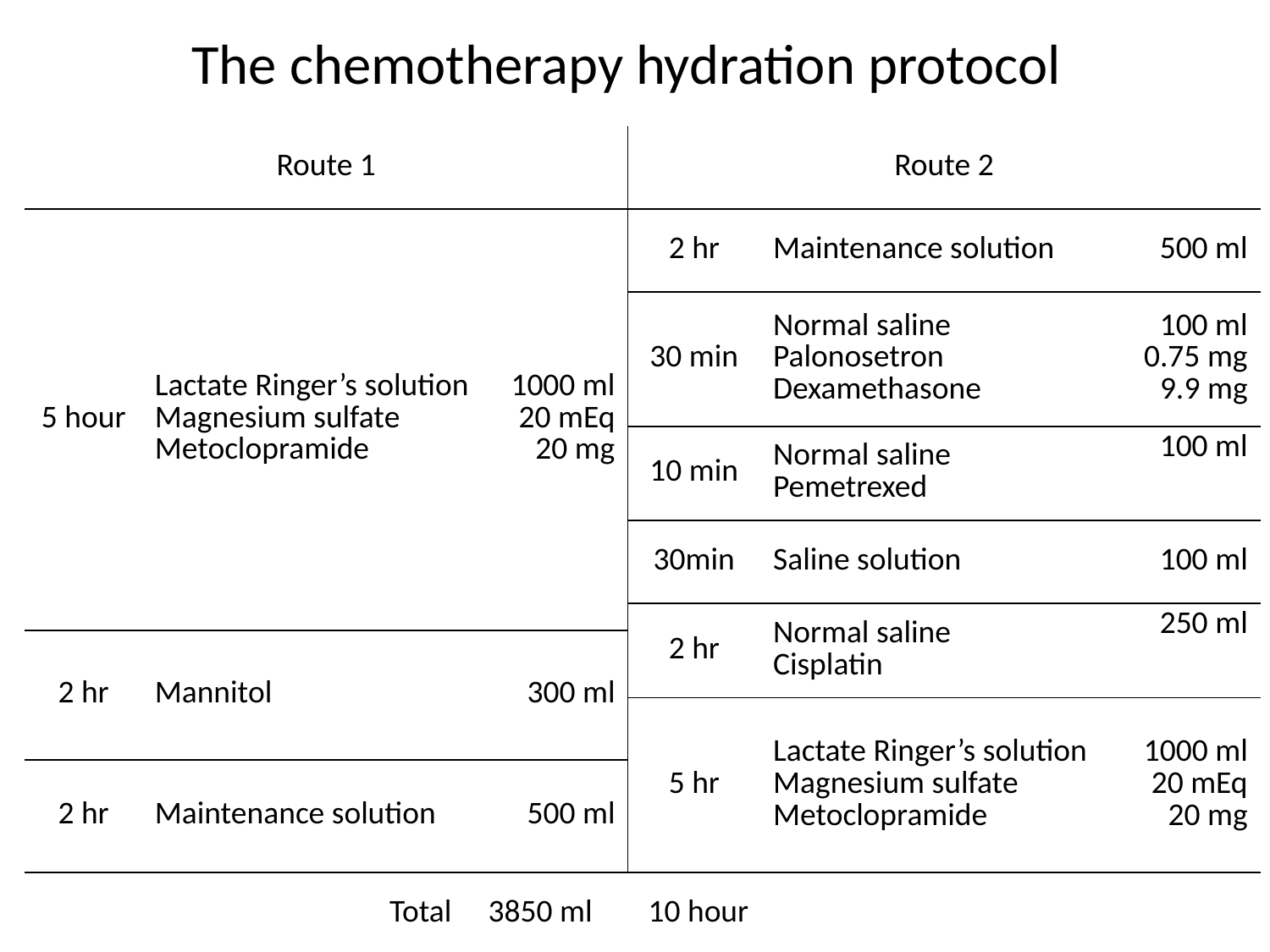

# The chemotherapy hydration protocol
| Route 1 | | | Route 2 | | |
| --- | --- | --- | --- | --- | --- |
| 5 hour | Lactate Ringer’s solution Magnesium sulfate Metoclopramide | 1000 ml 20 mEq 20 mg | 2 hr | Maintenance solution | 500 ml |
| | | | 30 min | Normal saline Palonosetron Dexamethasone | 100 ml 0.75 mg 9.9 mg |
| | | | 10 min | Normal saline Pemetrexed | 100 ml |
| | | | 30min | Saline solution | 100 ml |
| | | | 2 hr | Normal saline Cisplatin | 250 ml |
| 2 hr | Mannitol | 300 ml | | | |
| | | | 5 hr | Lactate Ringer’s solution Magnesium sulfate Metoclopramide | 1000 ml 20 mEq 20 mg |
| 2 hr | Maintenance solution | 500 ml | | | |
Total
3850 ml
10 hour
